# Editorial: Targeting cancer cell intracellular metabolism as a strategy against therapy resistance

**DOI:** 10.3389/fonc.2023.1144821

**Published:** 2023-02-15

**Authors:** Lingling Liu, Xiaohong Cai, Yuan Zhou, Chengsi He, Hongsheng Li, Xiangfu Liu, Zhengzhi Zou

**Affiliations:** ^1^ Department of Hematology, The Third Affiliated Hospital of Sun Yat-sen University & Sun Yat-sen Institute of Hematology, Guangzhou, China; ^2^ MOE Key Laboratory of Laser Life Science & Institute of Laser Life Science, College of Biophotonics, South China Normal University, Guangzhou, China; ^3^ Department of Breast Surgery, Affiliated Cancer Hospital & Institute of Guangzhou Medical University, Guangzhou, China; ^4^ Department of Blood Transfusion, the Third Affiliated Hospital, Sun Yat-sen University, Guangzhou, China

**Keywords:** cancer metabolism, drug resistance, cancer treatment, immune infiltration, tumor prognosis

Convincing evidence has revealed that cancer metabolism plays an important role in the occurrence and development of tumors. Abnormal cancer cell metabolism not only induces tumor proliferation, migration, and invasion but also inhibits immune infiltration. Hence, the intervention of tumor metabolic pathways is a novel treatment for cancer therapy. This Research Topic highlights recent findings on cancer metabolism and elucidates the underlying mechanism of several genes and a new diet in different cancers, as well as provides new strategies to overcome the development of drug resistance.

Although more than 200 genes are related to the prognosis of liver cancer, there are not enough genes that can be used as biomarkers and therapeutic targets. Wei et al. excavated data from TIMER and GEPIA databases to explore the role of RRP12 in hepatocellular carcinoma (HCC). The research is the first to report that RRP12 is upregulated in cancer cells and is associated with poor prognosis in HCC. Moreover, this study shows that RRP12-induced metabolism and immune escape are associated with tumor progression and poor prognosis. At last, suppression of RRP12 expression inhibits proliferation, invasion, and migration of the HCC cell lines. It indicates that RRP12 may be a potential target and prognostic biomarker for the treatment of HCC.

Although the liver is the center of ketone body (KB) production, the specific effects of the ketogenic diet (KD) on HCC are unclear. Thus, Lan et al. conduct a review of the metabolism of KBs from basic studies to clinical trials. First, this review introduces the generation and metabolism of KB in the liver as well as the component of the classical and improved KD. The potential effects of the KD on HCC are then investigated in terms of glucose metabolism, lipid metabolism, and immune response. According to studies, the KD can enhance the anti-tumor effect of PI3K inhibitors by inhibiting the insulin-induced mTOR pathway and exert anti-HCC effects by increasing the expression of HMGCS2 that is negatively associated with the tumor. Furthermore, β-OHB, the main component of KBs, may exert anti-inflammatory effects by activating GPR109A and inhibiting the NF-κB pathway. However, pre-clinical and clinical trials show that only early dietary intervention with KD has an anti-tumor effect, and side effects should be paid attention to in future studies to ensure the safety and feasibility of the KD. Therefore, the review is expected to stimulate new research to explore whether the combination of the KD and other anti-cancer treatments can enhance the therapeutic effect of cancer.

The study conducted by Guo et al. intends to explore the prognostic value of SOAT1 and its relationship with immune infiltration using GEPIA and TIMER2.0 databases. According to their research, SOAT1 expression being associated with poor prognosis in gliomas may be related to immune infiltration in the tumor microenvironment (TME). The study also demonstrates that SOAT1 overexpression may increase the secretion of chemokines to attract immune cell infiltration within the TME. Additionally, Guo et al. attempt to find out the function of SOAT1 in glioma pathogenesis. Their research demonstrates that SOAT1 participates in numerous biological functions by regulating several signaling pathways throughout the etiology of glioma. The study implies that SOAT1 may serve as a novel target in gliomas.

The DNA-dependent protein kinase catalytic subunit (DNA-PKcs) plays an important role in DNA damage and genome integrity. Furthermore, recent studies have revealed the critical functions of DNA-PK in tumor progression and therapeutic response, referring to its role in transcription regulation. However, the complex transcriptome dependent on DNA-PK is unknown. Therefore, Song et al. introduce long-read sequencing-based technology to study the transcriptome after DNA-PK inactivation. Their study identifies a large number of novel transcripts that have been validated by exon-specific PCR. Surprisingly, this long-read sequencing-based technology collects more information about gene transcription than previous methods. This study provides theoretical support for further research on the targeting of DNA-PK in human malignancies, as well as insights into potential underlying mechanisms of DNA-PK.

Shu et al. profile osteosarcoma (OS) subtypes based on platelet-related genes (PRGs) using TCGA and GEO databases. According to the study, the OS can be distinguished into two subtypes, C1 and C2, based on PRGs. The C1 subtype with more immune cells has a superior prognosis than the C2 subtype. Furthermore, when compared with the C1 subtype, 169 differentially expressed genes (DEGs) and pathways are found to be downregulated in the C2 subtype. It is noteworthy that the results indicate that platelet score according to the expression of the selected six genes is negatively associated with the infiltration of immune cells and the prognosis. Therefore, this study brings an expectation for OS prediction and therapeutic drug development based on platelet characteristic DEGs.

The study conducted by Chen et al. aims to explore the underlying mechanism of BTN3A3 in the progression of ovarian cancer. This study demonstrates that BTN3A3 is a good prognosis marker, and can bind to FGF2 to reduce the protein level of FGF2, and then in turn decrease the phosphorylation level of ERK1/2. So, the inhibitor of ERK1/2 is used to suppress the proliferation, migration, and invasion of the cancer cells with BTN3A3 knockdown. Their findings confirm the role of BTN3A3 in breast cancer and suggest that it may be a potential therapeutic target for ovarian cancer.

## Author contributions

LL and XC reviewed the literature and drafted the manuscript. HL, XL, and ZZ reviewed and edited the manuscript. YZ and CH reviewed and revised the manuscript and all authors approved the submission.

